# The Janus Face of SPAG6: Inducing EMT in Luminal Breast Cancer Cells Amidst Widespread Expression Loss in Breast Tumours

**DOI:** 10.1111/jcmm.70870

**Published:** 2025-10-09

**Authors:** Antonio Sechi, Jolein Mijnes, Sophia Villwock, Michael Rose, Florian Steib, Sarah Bringezu, Jonas Berger, Carmen Schalla, Sonja von Serenyi, Jana Dietrich, Nadina Ortiz‐Brüchle, Lara Heij, Jan Bednarsch, Oleg Gluz, Ulrike Nitz, Nadia Harbeck, Monika Graeser, Christine zu Eulenburg, Mohammad Parsa Mohammadian, Katarzyna Jóźwiak, Hans‐Heinrich Kreipe, Matthias Christgen, Martin Radner, Danny Jonigk, Edgar Dahl

**Affiliations:** ^1^ Department of Cell and Tumor Biology, Medical Faculty RWTH Aachen University Aachen Germany; ^2^ Institute of Pathology, Medical Faculty RWTH Aachen University Aachen Germany; ^3^ Center for Integrated Oncology Aachen Bonn Cologne Düsseldorf (CIO ABCD) Aachen Germany; ^4^ Institute of Pathology, University Hospital University of Ulm Ulm Germany; ^5^ Institute of Pathology, University Hospital of Essen Essen Germany; ^6^ Department of Surgery and Transplantation University Hospital Essen Essen Germany; ^7^ Breast Center Niederrhein, Evangelical Hospital Bethesda Moenchengladbach Germany; ^8^ Women's Clinic and Breast Center University Clinics Cologne Cologne Germany; ^9^ West German Study Group Moenchengladbach Germany; ^10^ Breast Center, Department of Gynecology and Obstetrics and CCC Munich LMU University Hospital Munich Germany; ^11^ Department of Gynecology University Medical Center Hamburg Hamburg Germany; ^12^ Institute of Biostatistics and Registry Research Brandenburg Medical School Theodor Fontane Neuruppin Germany; ^13^ Institute of Pathology, Medical School Hannover Hannover Germany; ^14^ German Center for Lung Research (DZL) BREATH Hannover Germany

**Keywords:** breast cancer, cell migration, EMT, luminal A, SPAG6

## Abstract

Understanding the role and molecular regulation of genes associated with tumour cell motility may be informative for future cancer therapy development. *Sperm‐associated antigen 6* (*SPAG6*) gene, encoding an evolutionarily highly conserved flagellar motility protein, is regulated by promoter hypermethylation in breast cancer. Our in silico analysis of healthy and breast cancer tissues from The Cancer Genome Atlas (TCGA) showed tumour‐specific *SPAG6* promoter hypermethylation in all molecular subtypes. Immunohistochemistry on the independent WSG PlanB breast cancer cohort (*n* = 2241) confirmed comprehensive down‐regulation of SPAG6 on the protein level. In vitro models demonstrated that SPAG6 overexpression in luminal cells exhibited strongly increased migration capacity (*p* < 0.0001) and characteristics of epithelial‐mesenchymal transition (EMT) with actin and E‐cadherin displacement. We propose that SPAG6 may have an important role in triggering the EMT program in luminal breast cancer cells, driving tumour progression and metastasis.

**Trial Registration:** ClinicalTrials.gov identifier: NCT01049425

## 1 Introduction

Breast cancer remains the most frequently diagnosed cancer and a leading cause of cancer deaths among women worldwide. Approximately 2.1 million women are newly diagnosed with this disease every year, accounting for a quarter of all cancers in women [1]. Breast cancer is a very heterogeneous disease with diverse histopathological, genetic and epigenetic characteristics, being associated with different clinical outcomes [2, 3]. Recent studies have shown that *de novo* promoter hypermethylation is both an early and frequent event in breast cancer development and that it is also involved in tumour progression [4, 5, 6, 7]. DNA methylation takes place at the 5‐carbon position of cytosines located 5′ to guanine, the so‐called CpG dinucleotide (CpG) [8]. CpGs cluster into CpG islands at promoter regions of approximately 50% of human genes [8, 9, 10]. In breast cancer, hypermethylation of CpG islands in promoter regions is frequently observed [8, 9, 10] being an important mechanism for tumour suppressor gene (TSG) inactivation [8, 10, 11, 12]. Epigenetically inactivated TSGs are also called class 2 TSGs to distinguish them from TSGs that have genetic alterations and represent class 1 TSGs [13, 14]. Potential class 2 TSGs reported to be hypermethylated in breast cancer play important roles in e.g., cell‐cycle regulation, apoptosis, DNA repair, tissue invasion and metastasis, angiogenesis and hormone signalling [6, 15]. Analysing cancer‐specific promoter hypermethylation of class 2 TSGs and understanding their contribution to breast carcinogenesis may give important clues towards more precise breast cancer prognosis and treatment [15].

We previously found the gene *Sperm Associated Antigen 6* (*SPAG6*) to be hypermethylated in breast cancer and characterised it as part of the SNiPER panel for liquid biopsy‐based early breast cancer detection [16]. *SPAG6* is an orthologue of *Chlamydomonas reinhardtii* paralysed flagella (*PF16*), which encodes for a protein localised to the central apparatus of the 9 + 2 axoneme involved in cilia and flagella motility [17, 18, 19, 20]. SPAG6 contains eight continuous armadillo repeats which are involved in protein–protein interactions [18, 20, 21, 22]. Approximately half of SPAG6 deficient mice die from hydrocephalus before adulthood, and males surviving to maturity are infertile due to reduced flagellar motility [18, 19]. Interestingly, SPAG6 knockout mice revealed improper formation of the immunological synapse due to the loss of centrosome polarisation and actin clearance, resulting in impaired humoral immunity [23]. A study on SPAG6 overexpressing COS‐1 cells revealed that SPAG6 localised to structures that were positive for tubulin [21]. Also, Zhang et al. found that SPAG6 decorates a subset of microtubules [24], especially those surrounding the nucleus [25].

Our original hypothesis was that *SPAG6* functions, in general, as a class 2 tumour suppressor gene (which we abbreviate C2TSG, [26]) due to its frequent hypermethylation in breast cancer [16] and our extended analysis below. A similar situation has been described in lung cancer (NSCLC) where SPAG6 expression is also frequently lost due to promoter hypermethylation [27]. However, recent data from other proliferative diseases have assigned an oncogenic role to SPAG6, e.g., in myelodysplastic syndromes (MDS) and acute myelogenous leukaemia (AML), where it is found to be overexpressed [28, 29]. In AML patients, SPAG6 expression could be used alongside six other genes to sensitively monitor minimal residual disease and prognosis [29, 30]. Silencing of *SPAG6* in MDS cell line SKM‐1 and AML cell line K562 resulted in a significant decrease in proliferation and an increase in apoptosis, which was accompanied by increased expression of TP53, PTEN and several caspases, suggesting regulation of apoptosis by the PI3K/Akt pathway [28]. In addition, tumours from liver cancer patients showed significantly higher SPAG6 expression compared to normal liver tissue, and abundant tumour expression was associated with a lower 5‐year survival rate [31]. Thus, SPAG6 appears to have different functions (i.e., tumour‐suppressive or oncogenic functions) depending on the tissue type and the given molecular context of the cells. To date, the expression and function of SPAG6 in breast cancer have not been studied. We therefore aimed to investigate its role in this multifaceted and heterogeneous disease. Our data show clear evidence for a role of SPAG6 in EMT and migration in luminal breast cancer cells.

## 2 Methods

Additional methods can be found in the online S1.

### 2.1 Cell Lines

Human breast cancer cell lines T‐47D (RRID:CVCL_0553) and MCF‐7 (RRID:CVCL_0031) were obtained from the American Type Culture Collection (ATCC, Rockville, MD, USA) and cultured according to the manufacturer's instructions (37°C, 95% humidity, 5% CO_2_). The cell lines were tested for the presence of mycoplasma every month and authenticated at the start of this work using the Multiplex Cell Authentication by Multiplexion GmbH (Heidelberg, Germany). All experiments were performed with mycoplasma‐free cells.

### 2.2 Cloning of SPAG6‐EGFP

The coding sequence for SPAG6 (transcript variant 1, NM_012443) was PCR amplified from the SPAG6‐pT‐Rex‐DEST30 vector (Source BioScience, Nottingham, United Kingdom) and cloned into the pWPXL‐EGFP expression vector (D. Trono, École Polytechnique Fédérale de Lausanne, Lausanne, Switzerland) using BamHI and MluI to generate pWPXL‐SPAG6‐EGFP (Table S10). The correct sequence of SPAG6‐EGFP was confirmed by DNA sequencing.

### 2.3 Cell Proliferation and XTT Assays

Cell proliferation was determined at 24, 48, 72 and 96 h after seeding 1 × 10^5^ cells/well in 6‐well plates. All measurements were done in triplicate. At the chosen time points, cells were counted with a CASY cell counter and analysed (OMNI Life Science, Bremen, Germany). For XTT assays, 1 × 10^3^ cells/well were plated in a 96‐well plate. Every 24 h, cell viability was determined using the XTT cell proliferation kit II (Roche Diagnostics, Mannheim, Germany) according to the manufacturer's instructions. Wells containing medium without cells served as the negative controls. Measurements were done with a Tecan Infinite 2000 ELISA reader (Tecan, Männedorf, Switzerland).

### 2.4 Apoptosis Assay

For this assay, 2 × 10^4^ cells/well were plated in a 96‐well plate, and after 24 h, apoptosis was quantified using the Apo‐ONE homogeneous caspase‐3/7 assay kit (Promega, Madison, USA) according to the manufacturer's instructions. Cells treated with staurosporin (1 μM) served as the positive controls. Fluorescence measurements were done using a Tecan Infinite 2000 ELISA reader (Tecan, Männedorf, Switzerland).

### 2.5 Colony Formation Assay

1 × 10^3^ cells were plated in triplicates in a 6‐well plate for up to two weeks. Colony formation was checked every day to determine the endpoint. Afterwards, cells were fixed and stained with 0.5% crystal violet (10% formaldehyde, 80% methanol and 10% H_2_O). The plates were then dried, imaged and the colonies counted manually.

### 2.6 Statistical Analysis

Descriptive statistics were calculated to summarise data. The normal distribution of numerical variables was checked using the D'Agostino‐Pearson normality test. A Mann–Whitney U test or t‐test was performed to compare two independent groups. One‐way ANOVA in combination with the Tukey method was used to compare three or more independent groups. Spearman's correlation was calculated to determine the association between *SPAG6* promoter methylation and gene expression. Using data from the phase III WSG PlanB trial, prognostic effects of SPAG6 on disease‐free (DFS) and overall survival (OS) were evaluated. Kaplan–Meier curves were plotted and compared with a log‐rank test between the categories of SPAG6. Univariable Cox models were built with all demographic and baseline variables as separate covariates. Additionally, bivariable Cox models were built with the categories of SPAG6 and each other covariate separately to identify confounders of the SPAG6 effect. A variable that changed the SPAG6 effect from an univariable Cox model by more than 10% was considered a confounder. Multivariable Cox models included categories of SPAG6, all confounders and all variables with *p* < 0.1 in univariable models. Non‐significant variables in the multivariable model were removed unless the SPAG6 effect changed by more than 10%. Moreover, the interaction between luminal A status and SPAG6 was tested in the final multivariable model. The proportionality assumption was tested using Schoenfeld residuals. *p*‐Values ≤ 0.05 were considered significant. Statistical analyses were performed with SPSS 25.0 (SPSS, Chicago, USA), GraphPad Prism 10.0 (GraphPad Software Inc., La Jolla, USA), R Statistical Software v4.0.3 (R Core Team, Auckland, New Zealand) and STATA version 17 (StataCorp. 2021. Stata Statistical Software: Release 17. College Station, TX: StataCorp LLC).

## 3 Results

### 3.1 Expression and Promoter Methylation of *SPAG6* in Breast Cancer Molecular Subtypes

**FIGURE 1:**
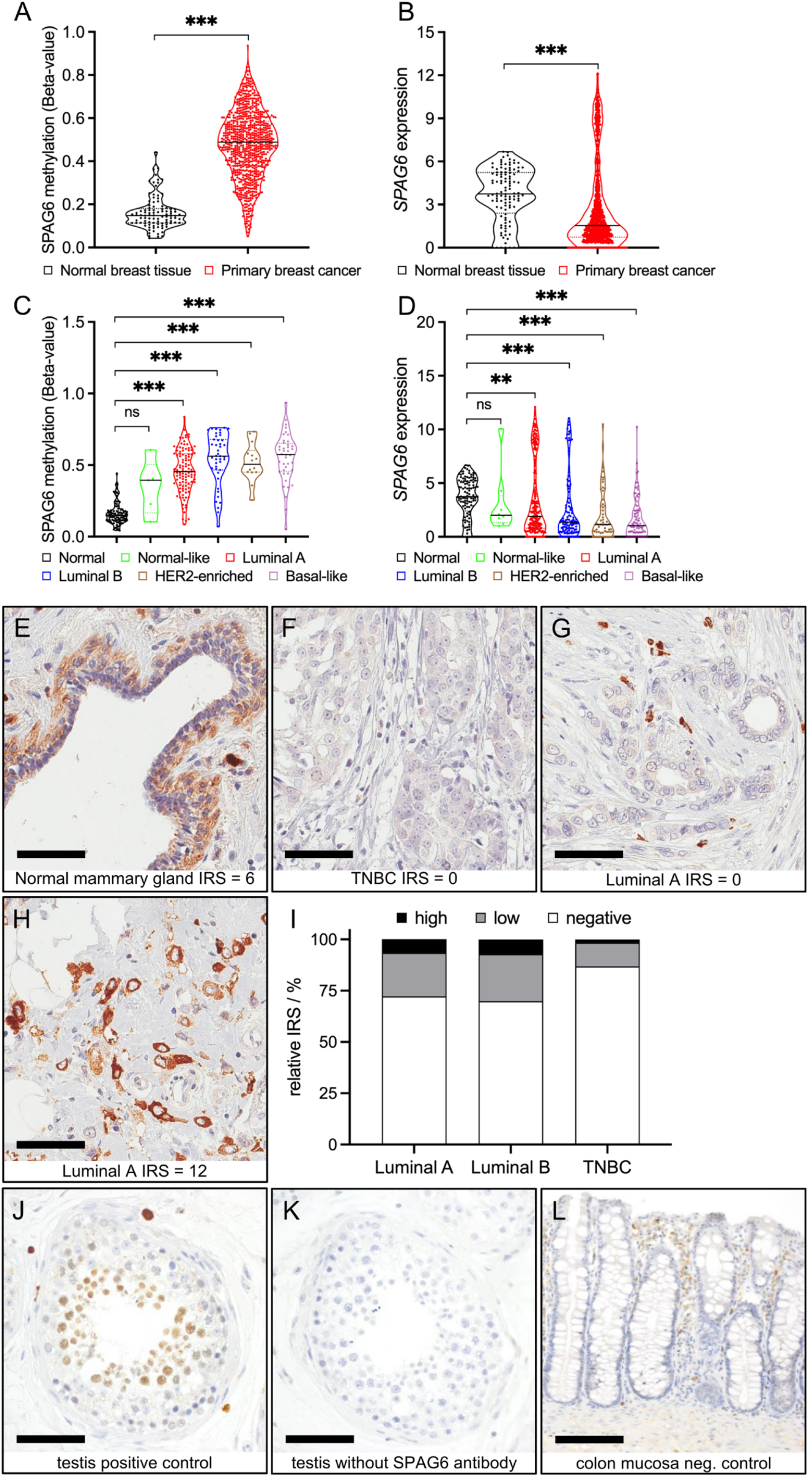
(A–D) Increase of *SPAG6* promoter methylation and decrease of *SPAG6* expression in breast cancer. In primary breast cancer, *SPAG6* promoter methylation is increased (A), while SPAG6 mRNA expression is decreased (B). The promoter methylation frequency is also increased in the different breast cancer subtypes (C). Similarly, the downregulation of *SPAG6* mRNA expression can be observed in all molecular breast cancer subtypes (D). Notice the large spread of the individual patients in both methylation level and gene expression. **p < 0.01, ***p < 0.001, ns, non‐significant. Lines in the boxes indicate the median. (E–L) Differential SPAG6 protein expression in human luminal‐type breast cancer. Paraffin sections were processed for immunohistochemical labelling according to standard protocols (see Materials and Methods for details) and stained with anti‐SPAG6 antibody. SPAG6 is abundant in normal mammary epithelium (E) but typically absent in TNBC (F). Luminal breast cancers showed variable SPAG6 expression: Most were negative or weak (G), while a small subset expressed SPAG6 abundantly (H). This high‐expressing subgroup was detectable in both luminal A and luminal B tumours, but not in TNBC (I). ‘Testis tissue showed strong SPAG6 in spermatocytes (J), absent staining without SPAG6 antibody (K). Colon mucosa, lacking endogenous SPAG6, served as negative control (L)’. Scale bars (E–L): 60 μm.

We previously detected *SPAG6* hypermethylation in the blood plasma of breast cancer patients, suggesting a possible role for this evolutionary highly conserved but poorly characterised gene in breast cancer biology [16]. Therefore, we sought to further investigate the expression level and methylation status of *SPAG6* in different types of breast cancer tissues from the TCGA breast cancer cohort. An overview of the clinical characteristics of this breast cancer cohort is summarised in Table S1. Consistent with our previous findings, normal breast tissue showed only low *SPAG6* promoter methylation (*β*‐value ≥ 0.2: 10.3%), which was significantly increased up to 95.7% in primary breast cancers (*p* < 0.001, Figure 1A). Conversely, *SPAG6* expression was significantly lower in breast cancer than in normal breast tissue (*p* < 0.001, Figure 1B). Moreover, *SPAG6* mRNA expression and DNA methylation were negatively and strongly correlated (Spearman *r*: −0.41, *p* < 0.0001). *SPAG6* hypermethylation was present in all molecular subtypes (classified by PAM50) except the normal‐like group (Figure 1C). Luminal A tumours showed the lowest mean methylation levels (mean *β*‐value: 0.46) compared to luminal B (mean *β*‐value: 0.54), HER2‐enriched (mean *β*‐value: 0.52) and basal‐like tumours (mean *β*‐value: 0.55). Interestingly, when looking at mRNA expression, a group of luminal A tumours (mean expression: 3.52; 75% percentile: 6.30—maximum: 12.10) and also a small group of luminal B tumours overexpressed *SPAG6* compared to healthy breast tissue (mean expression: 3.64; 75% percentile: 5.23—maximum: 6.68) as well as compared to basal‐like cancers (mean expression: 2.45; 75% percentile: 2.45—maximum: 10.26) (Figure 1D).

### 3.2 Comprehensive Protein Expression Analysis Confirms General SPAG6 Down‐Regulation in Breast Cancer, Coupled With Strong Upregulation in a Small Fraction of Luminal Tumours

SPAG6 staining data was available for 2241 of 3198 patients included in the phase III WSG PlanB trial. An overview of the clinicopathological parameters of these available patient data is shown in Table S2. While SPAG6 was abundantly expressed in normal breast tissue (Figure 1E), expression was almost completely lost in triple‐negative breast cancer (TNBC) (Figure 1F), confirming our previous conclusion that loss of SPAG6 expression due to promoter hypermethylation may be a suitable biomarker for detecting TNBC [16]. Interestingly, a differential expression pattern was found in luminal breast cancer (Figure 1G,H) that was not observed in TNBC (Figure 1I). Luminal tumours showed loss of SPAG6 expression in most cases, but a small group, approximately 10% of cases, showed high SPAG6 expression (Figure 1H for luminal A tumours), thus closely resembling the expression pattern found at the mRNA level (Figure 1D). As controls, testis tissue served as a positive control showing high endogenous SPAG6 protein expression in spermatocytes (Figure 1J), but not in samples where the SPAG6 antibody was omitted (Figure 1K). Additionally, colon mucosa tissue served as a negative control showing the absence of endogenous SPAG6 expression in epithelial cells (Figure 1L).

Next, the impact of loss of SPAG6 expression on survival was analysed. While typical prognostic markers such as nodal status, Ki67 status, and histological grade showed significant effects on OS and DFS, the effects of SPAG6 expression were not significant (Tables S3 and S4; Figure S1). The experimental treatment in the WSG PlanB study did not alter the estimated association between SPAG6 and outcome remarkably. These data suggest that loss of SPAG6 expression is not associated with an unfavourable prognosis in breast cancer, contrary to our original hypothesis based on the substantial SPAG6 promoter hypermethylation in this tumour entity. However, we observed an interesting trend in our dataset suggesting that SPAG6 may act as an oncogene in luminal A tumours. In the WSG PlanB cohort, the SPAG6 positive in comparison to SPAG6 negative tumours showed an increased risk of tumour recurrence (DFS) in luminal A tumours (HR 1.64, 95% CI 0.88–3.06) while the risk was decreased in luminal A‐negative tumours (0.86, 95% CI 0.58–1.28) (Tables S5 and S6). Although this effect did not yet reach statistical significance (*p* = 0.085 for the interaction), it could also be noticed in the TCGA cohort (Figure S2), albeit only in TP53‐mutated luminal tumours (Figure S2B,D), but not in TP53 wildtype luminal tumours (Figure S2A,C). Unfortunately, for the WSG‐PlanB cohort, a corresponding TP53 analysis could not be performed because the TP53 status was not available for this cohort.

### 3.3 SPAG6 Overexpression Significantly Affects the Colony Formation Capacity of Luminal‐Type Breast Cancer Cells

**FIGURE 2:**
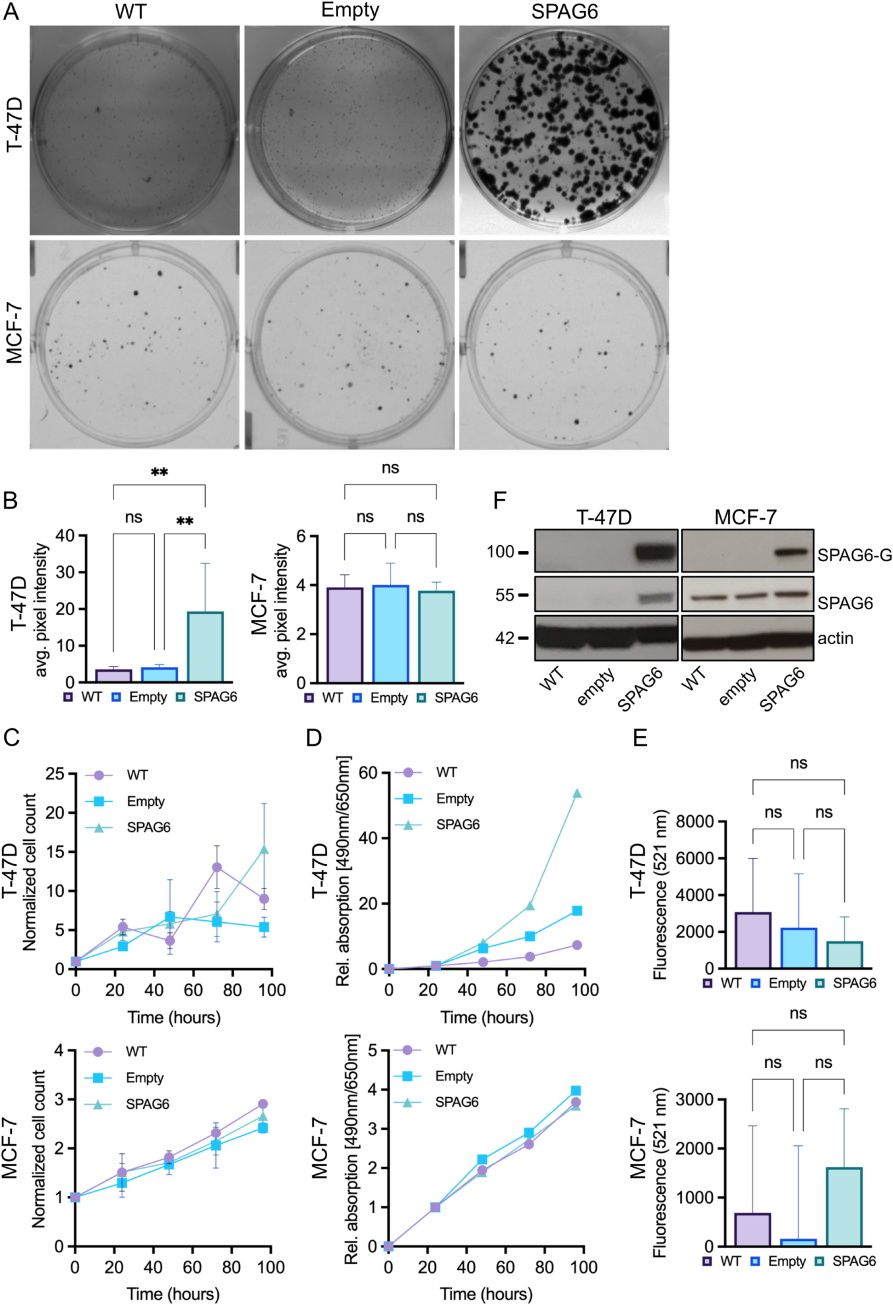
(A, B) SPAG6 promotes colony formation in T‐47D but not in MCF‐7 breast cancer cells. Control and SPAG6‐expressing cells were grown for 2 weeks for colony formation, then fixed and stained with crystal violet. (A) Representative images of colonies from WT, empty vector, and SPAG6‐overexpressing cells; note larger colonies in T‐47D upon SPAG6 overexpression. (B) Densitometry of stained colonies (mean ± SD) shows significantly increased colony formation in T‐47D with SPAG6 overexpression, whereas MCF‐7 cells were unaffected. **p < 0.01; ns, not significant. (C, D) SPAG6 promotes cell proliferation in T‐47D breast cancer cells. Cell proliferation assays (C) and XTT assays (D) show an increase in cell growth at later time points in T‐47D cells but not in MCF‐7 cells. Graphs show mean ± SD. (E) SPAG6 does not affect apoptosis in T‐47D and MCF‐7 cells. Bar charts show mean ± SD. ns, non‐significant. (F) Representative western blots showing the expression of endogenous SPAG6 and overexpresed SPAG6 in control T‐47D and MCF‐7 cells (WT), cells expressing the empty control vector and cells overexpressing SPAG6 (SPAG6‐GFP). 30 μg of total protein was loaded on each well and detected using the antibodies listed in Tables S8 and S9. As expected, overexpressed SPAG6 is robustly detectable in T‐47D and MCF‐7 cells. Notably, the expression of endogenous SPAG6 in T‐47D is increased in cells overexpressing SPAG6 (SPAG6‐GFP), whereas endogenous SPAG6 expression is not significantly affected in MCF‐7 under the same conditions. Note that despite loading equal amounts of total protein in each well, the levels of actin in each well slightly vary possibly due to SPAG6‐GFP overexpression.

Facing the interesting observation of the *SPAG6* mRNA expression pattern in the TCGA breast cancer data set and on the protein level in the large TMA breast cancer cohort, we aimed to decipher the role of SPAG6 in breast cancer gain‐of‐function cell culture models representing luminal (T‐47D and MCF‐7) breast cancer cell lines. To this end, these two cell lines were transduced with the SPAG6‐pWPXL vector encoding a SPAG6‐GFP fusion protein using a lentivirus‐based gene‐transfer system. Both SPAG6 mRNA and protein were robustly expressed in all transfected cell lines (Figure 2F).

First, the ability to form colonies was measured over a period of 14 days. Control T‐47D cells formed small but dense and easily distinguishable colonies, while cells overexpressing SPAG6 formed much larger colonies that were visible without a microscope (Figure 2A, upper panel). The quantification of mean grey scale values in colonies stained with crystal violet confirmed this observation (*p* = 0.0004, Figure 2B). However, modulation of SPAG6 expression in MCF‐7 breast cancer cells did not affect colony formation (Figure 2A, lower panel and 2B, *p* = 0.7304). SPAG6 overexpression also promoted cell proliferation in T‐47D breast cancer cells but not in MCF‐7 cells, as shown by cell proliferation assays and XTT assays (Figure 2C,D). SPAG6 overexpression did not affect apoptosis in both T‐47D and MCF‐7 cells (Figure 2E).

### 3.4 SPAG6 Significantly Promotes the Migration of Luminal‐Type T‐47D and MCF‐7 Breast Cancer Cells

**FIGURE 3:**
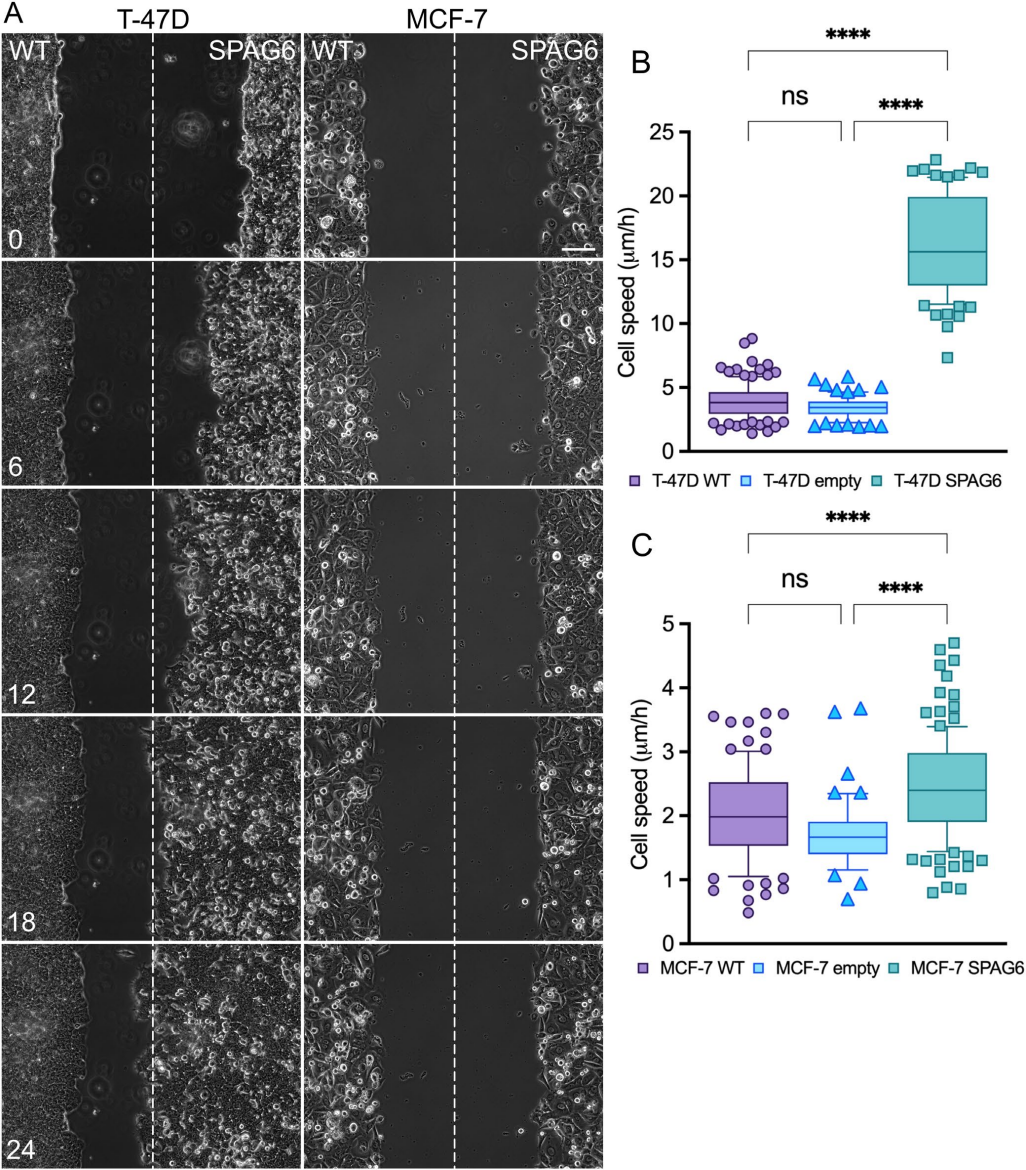
SPAG6 overexpression induces migration capacity of luminal‐type T‐47D and MCF‐7 breast cancer cells. A uniform scratch was introduced in confluent T‐47D and MCF‐7 cultures, and cell migration into the gap was monitored by time‐lapse microscopy. Average speed was calculated from the distance travelled by the cell front after 24 h (numbers in A indicate elapsed time). (A) Representative images of wound closure in control and SPAG6‐overexpressing cells; dashed line marks the middle of the scratch at time 0. SPAG6 overexpression markedly enhanced migration in T‐47D cells (B), but less significantly in MCF‐7 cells (C). Scale bar in A: 100 μm. (B+C) The empty vector had no effect. ****p < 0.0001.

Classical wound healing assays were performed to determine the effects of SPAG6 overexpression on the migration of luminal‐type breast cancer cell lines. The overexpression of SPAG6 in T‐47D cells robustly increased their migration capacity, as evidenced by a much faster wound closure compared with wild‐type cells (Figure 3A,B). In MCF‐7 cells overexpressing SPAG6, there was no gross difference in motility compared with wild‐type cells on a visual inspection (Figure 3A). Therefore, the average cell speed was quantified at six positions along each wound edge. T‐47D cells migrated with a median speed of 3.9 μm/h, while cells overexpressing SPAG6 moved at 15.6 μm/h, i.e., 4× faster (*p* < 0.0001, Figure 3B). Quantitative measurements further showed that MCF‐7 cells overexpressing SPAG6 also migrated significantly faster than their control counterparts (wild‐type cells median speed: 2.0 μm/h, SPAG6 overexpressing cells median speed: 2.4 μm/h, *p* < 0.0001, Figure 3C, see Videos S1 and S2). In both T‐47D and MCF‐7, the expression of the empty vector did not affect cell migration (Figure 3B,C). Overall, these data demonstrate that SPAG6 overexpression can significantly induce cell migration capacity in luminal‐type breast cancer cells.

### 3.5 SPAG6 Overexpression Induces EMT and Causes a Change in the Molecular Composition of Cell–Cell Junctions in T‐47D Breast Cancer Cells

**FIGURE 4:**
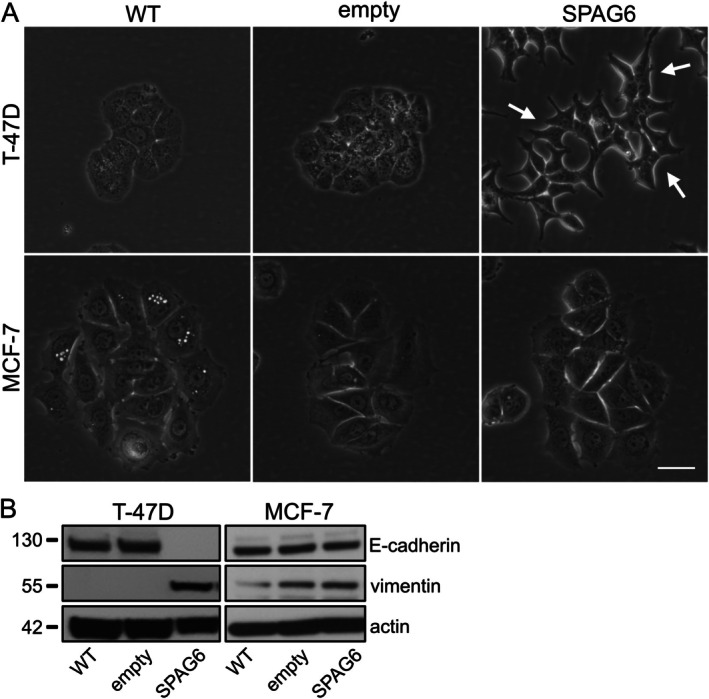
SPAG6 Induces morphological changes and regulates the expression of EMT genes in T‐47D breast cancer cells. Subconfluent T‐47D and MCF‐7 cultures were analysed 24 h after seeding by phase‐contrast microscopy at 37°C, 5% CO_2_, and constant humidity. T‐47D cells overexpressing SPAG6 showed a morphological shift from epithelial to mesenchymal (A, upper panel, white arrows), while MCF‐7 cells showed no major changes (A, lower panel). Scale bar: 100 μm. (B) Western blots confirm E‐cadherin downregulation and vimentin upregulation in SPAG6‐expressing T‐47D cells. Despite equal protein loading, actin levels varied slightly, possibly due to SPAG6‐overexpression.

**FIGURE 5:**
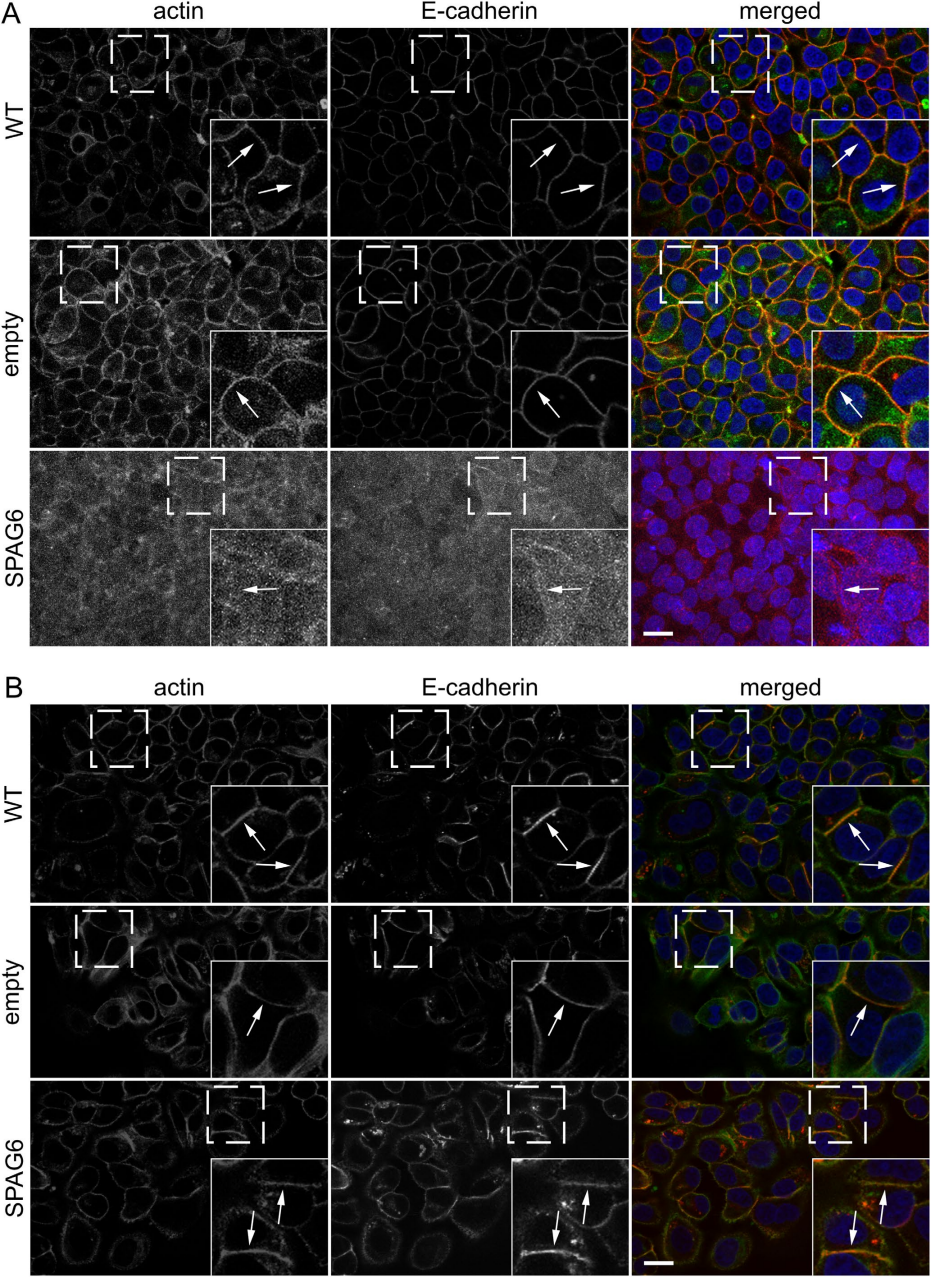
SPAG6 regulates cell–cell junction formation in T‐47D breast cancer cells. Cells grown on glass coverslips were fixed and stained for E‐cadherin (red), F‐actin (phalloidin, green), and nuclei (DAPI, blue). Representative images show actin and E‐cadherin co‐localisation at junctions in T‐47D (A) and MCF‐7 cells (B), both in WT and empty vector controls (arrows in insets). SPAG6 overexpression markedly reduced junctional actin and E‐cadherin only in T‐47D cells. Dashed boxes mark enlarged insets. Scale bars: 20 μm.

Overexpression of SPAG6 in T‐47D cells also induced striking morphological changes. For example, T‐47D cells overexpressing SPAG6 detached more readily from the surface of the cell culture vessel and formed lamellipodial protrusions that were absent in wild‐type cells (Figure 4A). We therefore hypothesised that SPAG6 might affect EMT in these cells. In T‐47D cells overexpressing SPAG6, the expression of E‐cadherin and vimentin, two major EMT markers, decreased and increased, respectively (Figure 4B). Consistent with the absence of gross morphological EMT changes in MCF‐7 cells overexpressing SPAG6, the expression of E‐cadherin and vimentin was not grossly affected by SPAG6 (Figure 4B). In T‐47D cells, overexpression of SPAG6 promoted the formation of less compact cell monolayers. To test the hypothesis of whether SPAG6 affects the formation of cell–cell junctions, we seeded T‐47D and MCF‐7 cells at high cell density and stained them after 24 h with antibodies against β‐catenin and E‐cadherin, two specific markers of cell–cell junctions. As expected in wild‐type control cells and cells expressing only the empty vector, E‐cadherin was strongly localised at cell–cell junctions, where it was found together with actin (Figure 5A). Of note, in T‐47D cells overexpressing SPAG6, both actin and E‐cadherin were barely found at cell–cell junctions (Figure 5A). In MCF‐7 cells overexpressing SPAG6, no changes in actin and E‐cadherin localisation were observed (Figure 5B).

**FIGURE 6:**
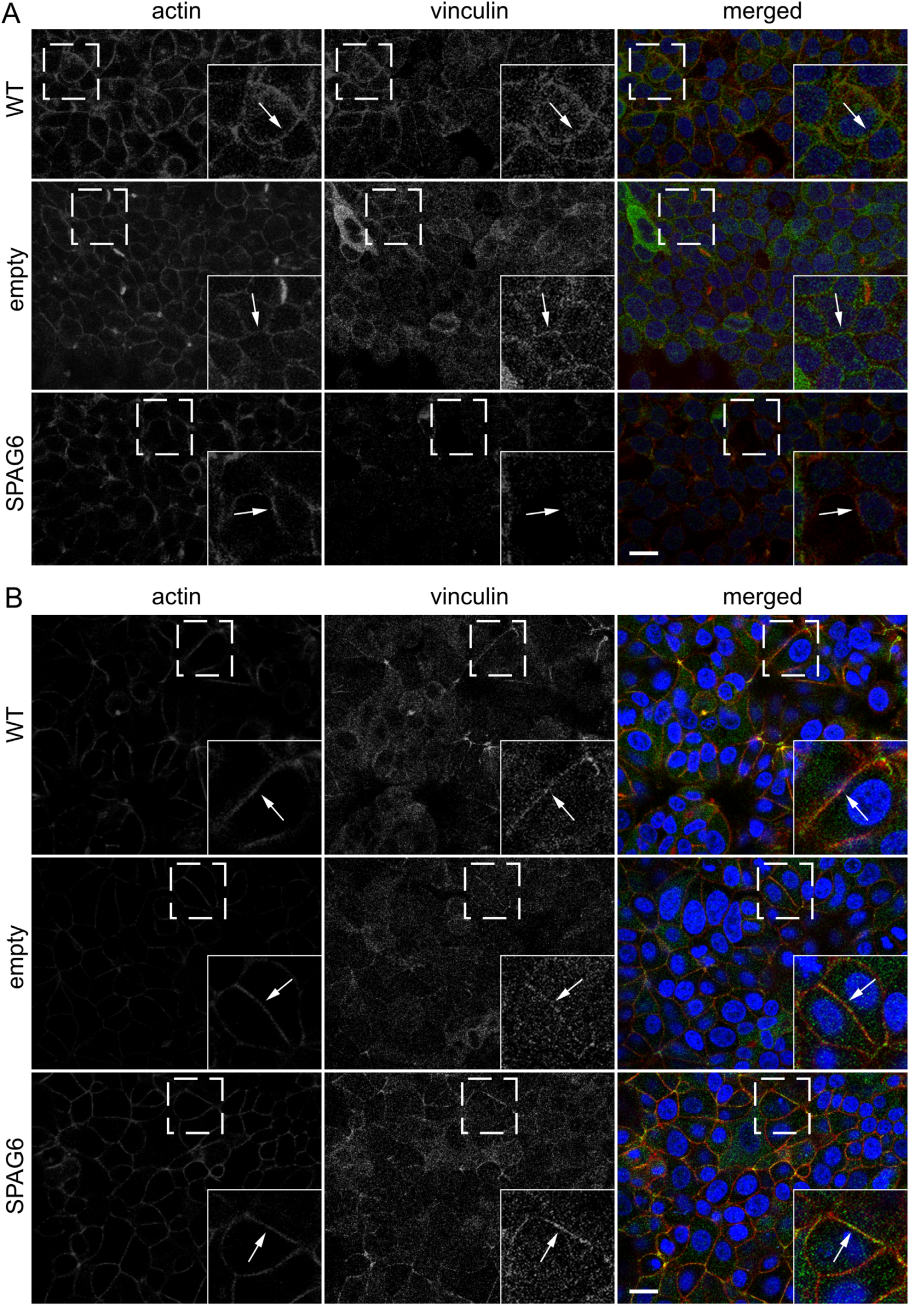
SPAG6‐overexpression suppresses actin and vinculin localisation at cell–cell junction in T‐47D breast cancer cells. Cells on glass coverslips were fixed and stained for vinculin (green), F‐actin (phalloidin, red), and nuclei (DAPI, blue). Representative images show actin–vinculin co‐localisation at junctions in T‐47D (A) and MCF‐7 cells (B) in WT and empty vector controls (arrows in insets). SPAG6 overexpression markedly reduced junctional actin and vinculin only in T‐47D cells. Dashed boxes indicate enlarged insets. Scale bars: 20 μm.

**FIGURE 7:**
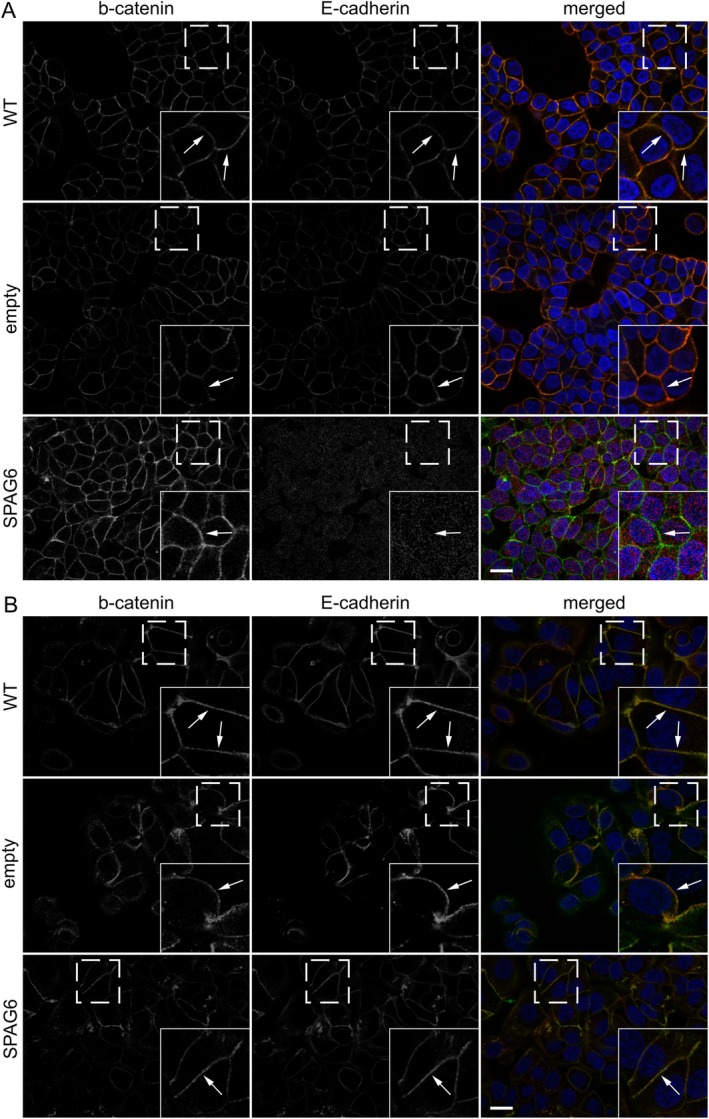
SPAG6 overexpression does not change ß‐catenin localisation at cell–cell junctions in T‐47D breast cancer cells. Cells on glass coverslips were fixed and stained for E‐cadherin (red), β‐catenin (green), and nuclei (DAPI, blue). Representative images show robust co‐localisation of β‐catenin and E‐cadherin at junctions in T‐47D (A) and MCF‐7 (B) WT and empty vector controls (arrows in insets). SPAG6 overexpression in T‐47D cells strongly reduced junctional E‐cadherin without affecting β‐catenin, while MCF‐7 cells remained unchanged. Dashed boxes mark enlarged insets. Scale bars: 20 μm.

Since vinculin interacts directly with actin and is localised at cell–cell junctions [32], we examined whether the reduction of actin at cell–cell junctions in cells expressing SPAG6 also affected the localisation of vinculin at these sites. Consistent with the above observations, expression of SPAG6 in T47D, but not in MCF‐7 cells, caused the displacement of vinculin and the reduction of actin at cell–cell junctions (Figure 6). Because E‐cadherin is typically associated with β‐catenin at cell–cell junctions [33, 34], we also labelled T‐47D and MCF‐7 cells with antibodies against β‐catenin and E‐cadherin. Interestingly, β‐catenin was found to localise at cell–cell junctions independent of SPAG6 expression (Figure 7A,B). These observations indicate that SPAG6 affects the formation and molecular composition of cell–cell junctions that correlate with an EMT phenotype in T‐47D breast cancer cells.

### 3.6 SPAG6 Overexpression Effect in Basal‐Type Breast Cancer Cell Lines Harbouring *TP53* Mutations

Due to the unexpected and interesting finding that SPAG6 may have tumour‐promoting properties in luminal breast cancer cells and that this oncogenic effect may be dependent on an inactivated *TP53* gene, we expanded our study to include the analysis of two basal breast cancer cell lines in which the *TP53* gene is mutated (Figure S3). In the MDA‐MB‐231 and BT‐549 breast cancer cell lines, SPAG6 overexpression led to inhibition of colony formation, while there was no significant effect on proliferation. Since both cell lines harbour *TP53* missense mutations, we conclude that the different biological behaviour of SPAG6 depends primarily on the molecular subtype (luminal versus basal) and only to a lesser extent on the *TP53* status.

## 4 Discussion

Understanding the function of novel candidate genes potentially involved in the development or progression of cancer is critical for exploring new ways to improve cancer diagnostics and therapy, particularly for personalised therapies. *SPAG6* is one such candidate gene whose function in motile unicellular organisms is quite well understood, but whose function in higher organisms is still largely unclear and has not yet been directly linked to cancer‐related processes. We recently identified the *SPAG6* gene as a novel potential class 2 tumour suppressor gene (C2TSG) that may be suitable for blood‐based breast cancer screening due to its frequent promoter DNA hypermethylation, particularly in basal‐type cancer [16]. This study is the first to show that SPAG6 has a profound influence on the migration and colony‐forming ability of luminal breast cancer cells, although future studies should expand the number of luminal cell lines examined. Moreover, SPAG6 induces EMT in T‐47D cells and has an impact on the formation and molecular composition of cell–cell junctions in this cell line.

In all molecular subtypes of breast cancer, except for the poorly defined ‘normal‐like’ group [35], the *SPAG6* promoter showed a significant increase in DNA methylation levels that was accompanied by a significant decrease in its expression. However, a closer look at the different molecular breast cancer subtypes luminal A and luminal B showed a relatively large scatter in both methylation frequency and SPAG6 mRNA expression. This was most pronounced in luminal A breast cancer, which was characterised by two distinct SPAG6 expression patterns, i.e., a large cluster with low expression and a small cluster with high expression (see 75th percentile in Figure 1D), the latter having higher SPAG6 mRNA expression than in normal breast tissue. Considering that for basal‐like breast cancer it has already been shown that this molecular subtype can be divided into several molecular ‘sub‐subtypes’ [36, 37], it would be desirable in the future to be able to subdivide the luminal A and luminal B breast cancer subtypes into further molecular subtypes, for which suitable biomarkers must be identified. We cannot yet claim that we have identified SPAG6 as such a biomarker, but the observation that the tumour‐promoting effect in luminal tumours might depend on the *TP53* mutation status is potentially interesting and should be followed up. So far, we have only analysed two luminal breast cancer cell lines and the one that is *TP53* mutant (T‐47D) shows significantly stronger tumour‐promoting effects after SPAG6 overexpression, such as EMT induction, than the *TP53* wild‐type cell line MCF‐7. Since mutated *TP53* is recognised for its role in enhancing the motility and metastasis of cancer cells, it is plausible that SPAG6 may synergise with mutated *TP53*, leading to robustly increased motility in T‐47D cells. In contrast, the effect (i.e., increase) of SPAG6 on the motility of MCF‐7 cells might be mitigated by wild‐type *TP53*, which is known for its ability to reduce cell motility. Currently, our hypothesis that luminal breast tumours with a *TP53* mutation and high SPAG6 expression may represent a more aggressive subtype can only be considered a noteworthy point for future SPAG6 expression studies, since the *TP53* status was not available for the PlanB cohort and the potential SPAG6‐induced oncogenic phenotype in the PlanB breast cancer cohort (HR 1.64, *p* = 0.085) did not reach statistical significance. This could be due to underpowering of the subgroup, as the proportion of SPAG6 ‘high expressers’ in luminal A and luminal B tumours accounted for only 5%–10% of all tumours in these subgroups. In such small groups, it is statistically difficult to validate the influence of other cofactors. Finally, the low number of tumours with high SPAG6 expression may also have led to insufficient significance of the survival data analysis. Ultimately, this problem can only be solved by analysing extremely large breast cancer cohorts for which the relevant histopathological information (markers for the classification of molecular subgroups, *TP53* mutation status, SPAG6 expression status) is available.

The hypothesis that the *SPAG6* gene may play different roles (i.e., potential tumour suppressor versus potential oncogene) in different cancer entities, or in different molecular contexts as defined by tumour morphological subgroups, is supported by the following data. The *SPAG6* promoter is hypermethylated and its expression is reduced in lung cancer (NSCLC) [27], suggesting a tumour‐suppressive function in this tumour entity. Hypermethylation of the *SPAG6* promoter was also found in non‐muscle invasive bladder cancer (NMIBC) [38, 39], although methylation did not appear to correlate with specific tumour characteristics [39]. In contrast, higher expression levels of SPAG6 have been demonstrated in haematological malignancies including lymphoma and myeloproliferative neoplasms (MPN) [40, 41, 42, 43]. AML patients with high *SPAG6* expression had poor survival. Further studies on AML and its precursor, myelodysplastic syndrome (MDS), suggested that SPAG6 regulates apoptosis through the PTEN/PI3K/AKT and TRAIL pathways [28, 44] and proliferation through the AKT/FOXO pathway [45] in both diseases. In MPN, the tumour‐promoting function of SPAG6 is associated with reduced response of MPN cells to interferon‐α treatment [46]. Recent research also indicates that SPAG6 enhances the proliferation of AML and B‐cell acute lymphoblastic leukaemia (B‐ALL) cells, as well as contributing to the growth of subcutaneous tumours [47, 48]. This suggests that SPAG6 could be a potential therapeutic oncogene‐like target in AML, B‐ALL and MDS. Liver cancer tissue also exhibited higher SPAG6 expression compared to healthy tissues, and patients with abundant *SPAG6* expression showed significantly lower 5‐year survival [31]. Moreover, the knockdown of *SPAG6* in the hepatocellular carcinoma cell line HCCLM3 resulted in impaired proliferation and migration, suggesting that SPAG6 may contribute to the development and progression of liver cancer [31]. The paradoxical dual role of SPAG6 as both a tumour promoter and a tumour suppressor depending on the tissue context cannot currently be explained yet. Nevertheless, this is not a new observation, as regulators that have been studied much more intensively (than SPAG6), such as Notch receptors and YAP (a terminal mediator of the Hippo signalling pathway), have been shown to have a dual role as growth promoters and inhibitors, depending on the molecular or cellular context [49, 50]. There are also some examples of well‐known tumour suppressor genes that can take on oncogenic functions, so this ‘double agent game’ of cancer‐associated genes remains an exciting field of research, which is also important for the clinical evaluation of genetic and epigenetic alterations [51]. What is known about SPAG6 protein is that it is a scaffolding protein that can engage in different signalling networks depending on the cellular context. The protein's eight armadillo repeats create multiple binding sites for diverse protein partners. Thus, SPAG6 could act as a molecular switch that either promotes or inhibits cancer progression depending on the available binding partners and dominant signalling pathways in a given cellular context. Interestingly, in some tumour entities, such as head and neck squamous cell carcinoma and lung squamous cell carcinoma, SPAG6 expression correlates with the expression of chemokines and major histocompatibility complex molecules, suggesting that immune cell‐derived signals may upregulate SPAG6 transcription [52].

Deregulation of cell motility and cytoskeleton function is a characteristic phenomenon in cancer cells that contributes to their dissemination to secondary sites [53, 54, 55]. During tumorigenesis, cancer cells undergo molecular and cellular changes that include remodelling of cell–cell adhesions, cell‐matrix adhesions, and deregulation of signalling pathways leading to remodelling of the actin cytoskeleton [56, 57, 58]. In this context, we demonstrated that SPAG6 has a robust effect on both T‐47D and MCF‐7 and significantly increases their motility. Our observations are consistent with previous studies showing that silencing of SPAG6 in the hepatocellular carcinoma cell line HCCLM3 impaired cell migration [18, 31]. Again, the cellular context and tumour type must be considered when examining SPAG6 function, as overexpression of SPAG6 could also have a suppressive effect, as shown in neuronal cells where it impairs cell migration [59, 60, 61]. Cell migration is a cyclic process established and maintained by signalling through Rho family GTPases, PI3Ks, integrins, microtubules and vesicular transport [62]. Although understanding the molecular mechanisms underlying SPAG6 function in cell migration is beyond the scope of this study, it is reasonable to hypothesize that SPAG6 may be involved either directly or indirectly in one or more of the above processes. For example, SPAG6 might affect microfilament, microtubule, and intermediate filament function via its eight contiguous Armadillo repeats [20], as previously shown for other proteins with Armadillo repeats, including β‐catenin [63, 64, 65, 66]. In addition to its interaction with microtubules, β‐catenin is a key node in Wnt signalling and a component of actin‐containing junctions that connect cells via cadherin proteins [66]. Finally, it is interesting to note that SPAG6 interacts with myosin 1D, a class I molecular motor that promotes colorectal and breast carcinogenesis and stimulates EGF receptor family signalling [42, 67]. Since myosin 1D and EGFR are known to promote cell migration [68], it is reasonable that SPAG6 regulates cell migration by binding to myosin 1D.

EMT is a biological process during which a cell within a tumour tissue undergoes several changes that enable it to acquire a mesenchymal cell phenotype associated with changes in migratory capacity, invasiveness, and increased resistance to apoptosis [57, 58, 69]. In our study, we demonstrate that SPAG6 overexpression induces several EMT characteristics in T‐47D cells. SPAG6 had a major effect on cell–cell adhesions characterised by the downregulation of E‐cadherin and reduction of actin, vinculin and β‐catenin localisation at these sites. Moreover, E‐cadherin and vimentin were upregulated by SPAG6 in T‐47D cells. It should be noted that SPAG6‐overexpressing MCF‐7 cells did not exhibit such a distinct EMT phenotype. This could be due to the wild‐type status of *TP53* in MCF7 cells, since SPAG6 could interact with different sets of proteins, depending on whether *TP53* is mutated or not. However, we cannot exclude that other differences (in addition to *TP53* mutations) between the two cell lines contributed to their behaviours. In this context, it is essential to highlight that SPAG6 overexpression is more pronounced in T‐47D cells, while MCF‐7 cells exhibit elevated levels of endogenous SPAG6. Consequently, the existing endogenous SPAG6 in MCF‐7 cells may render them ‘refractory’ to the expression of SPAG6‐GFP, leading to a more subdued alteration in behaviour. Since cancer cells typically exhibit different ‘stages’ of EMT, initially retaining some epithelial features while acquiring mesenchymal traits [57], the different effects of SPAG6 on T‐47D and MCF‐7 cells could reflect a more complete stage in T‐47D cells and a partial EMT stage in MCF‐7 cells.

The SPAG6 protein may emerge as a context‐dependent therapeutic target molecule in the near future. In cancers such as multiple myeloma, acute myeloid leukaemia and Burkitt's lymphoma, SPAG6 promotes proliferation, inhibits apoptosis and enhances tumour progression via important signalling pathways such as MAPK/ERK and PTEN/PI3K/AKT [70]. Thus, in addition to the direct inhibition of SPAG6 by gene silencing, small molecules or RNA‐based therapeutics, the inhibition of the above‐mentioned SPAG6‐activated signalling pathways could also represent an indirect therapeutic approach in tumour entities in which SPAG6 is strongly upregulated. Since SPAG6 modulates the immunological microenvironment by affecting tumour immune infiltration and interacting with immune checkpoint pathways, as described above, a third application for a SPAG6 inhibitor could be to overcome immune resistance.

Overall, we demonstrate that overexpression of SPAG6 regulates colony formation, migration capacity and intercellular interaction of luminal breast cancer cells. The impact of SPAG6 on cell migration and cell–cell junction formation suggests that this protein may play a role in inducing EMT in a distinct group of luminal breast cancer cells, potentially those that exhibit *TP53* mutation and abundant SPAG6 expression. In follow‐up studies, it will be interesting to determine in large luminal breast cancer cohorts whether abundant expression of SPAG6 correlates with metastasis and poor survival outcomes, taking into account the status of *TP53* mutations and other potential biomarkers. Future functional studies should focus on unravelling the molecular mechanisms underlying SPAG6‐driven regulation of cell migration and intercellular interactions to identify novel targets for the treatment of advanced luminal‐type breast cancer.

## Author Contributions

**Antonio Sechi:** conceptualization (equal), project administration (equal), supervision (equal), visualization (equal), writing – original draft (equal), writing – review and editing (equal). **Jolein Mijnes:** data curation (equal), investigation (equal), methodology (equal), visualization (equal), writing – original draft (equal). **Sophia Villwock:** data curation (equal), methodology (equal), visualization (equal). **Michael Rose:** data curation (equal), methodology (equal), visualization (equal). **Florian Steib:** investigation (equal), methodology (equal). **Sarah Bringezu:** investigation (equal). **Jonas Berger:** investigation (equal). **Carmen Schalla:** investigation (equal). **Sonja von Serenyi:** investigation (equal). **Jana Dietrich:** investigation (equal), visualization (equal). **Nadina Ortiz‐Brüchle:** resources (equal). **Lara Heij:** resources (equal). **Jan Bednarsch:** resources (equal). **Oleg Gluz:** validation (equal). **Ulrike Nitz:** validation (equal). **Nadia Harbeck:** validation (equal). **Monika Graeser:** validation (equal). **Christine zu Eulenburg:** formal analysis (equal), validation (equal), visualization (equal). **Mohammad Parsa Mohammadian:** formal analysis (equal), software (equal), validation (equal), visualization (equal). **Katarzyna Jóźwiak:** formal analysis (equal), software (equal), validation (equal), visualization (equal). **Hans‐Heinrich Kreipe:** validation (equal). **Matthias Christgen:** validation (equal). **Martin Radner:** validation (equal). **Danny Jonigk:** resources (equal). **Edgar Dahl:** conceptualization (equal), funding acquisition (equal), project administration (equal), supervision (equal), writing – original draft (equal), writing – review and editing (equal).

## Ethics Statement

The phase III planB study was approved by German ethics committees and conducted in accordance with the Declaration of Helsinki. The institutional review board (competent authority) was the BfArM (Federal Institute for Drugs and Medical Devices); the approval date was October 30, 2008. Informed consent was granted by the Central Ethics Committee, Ärztekammer Nordrhein, Tersteegenstrasse 9, D‐40474 Düsseldorf, Germany; the approval date was December 23, 2008. Animal testing is not applicable.

## Conflicts of Interest

The authors declare no conflicts of interest.

## Supporting information


**Data S1:** jcmm70870‐sup‐0001‐Supinfo.docx.

**Video S1:** jcmm70870‐sup‐0002‐VideoS1.avi.

**Video S2:** jcmm70870‐sup‐0003‐VideoS2.avi.

## Data Availability

The data that support the findings of this study are available from the corresponding author upon reasonable request.
