# Supplementary material for: The Janus Face of SPAG6: Inducing EMT in Luminal Breast Cancer Cells Amidst Widespread Expression Loss in Breast Tumours
Source: J Cell Mol Med. 2025 Oct 9;29(19):e70870. doi: 10.1111/jcmm.70870 (PMC12510430; doi:10.1111/jcmm.70870)
Supplement: Supplementary file 1 — Data S1: jcmm70870‐sup‐0001‐Supinfo.docx. [file JCMM-29-e70870-s003.docx]

**Supplementary information**

**The Janus face of SPAG6: Inducing EMT in luminal breast cancer cells amidst widespread expression loss in breast tumors**

Antonio Sechi^1¥*^, Jolein Mijnes^2,3¥^, Sophia Villwock^2,3^, Michael Rose^2,3,4^, Florian Steib^2,3^, Sarah Bringezu^2,3^, Jonas Berger^2,3^, Carmen Schalla^1^, Sonja von Serenyi^2,3^, Jana Dietrich^2,3^, Nadina Ortiz-Brüchle^2,3^, Lara Heij^5^, Jan Bednarsch^6^, Oleg Gluz^7,8,9^, Ulrike Nitz^7,9^, Nadia Harbeck^9,10^, Monika Graeser^7,9,11^, Christine zu Eulenburg^9^, Mohammad Parsa Mohammadian^12^, Katarzyna Jóźwiak^12^, Hans Heinrich Kreipe^13^, Matthias Christgen^13^, Martin Radner^13^, Danny Jonigk^2,3,14^, Edgar Dahl^2,3*^

^1^Department of Cell and Tumor Biology, Medical Faculty, RWTH Aachen University, Aachen, Germany

^2^Institute of Pathology, Medical Faculty, RWTH Aachen University, Aachen, Germany

^3^Center for Integrated Oncology Aachen Bonn Cologne Düsseldorf (CIO ABCD)

^4^Institute of Pathology, University Hospital, University of Ulm, Ulm, Germany

^5^Institute of Pathology, University Hospital of Essen, Essen, Germany

^6^Department of Surgery and Transplantation, University Hospital Essen, Essen, Germany

^7^Breast Center Niederrhein, Evangelical Hospital Bethesda, Moenchengladbach, Germany

^7^University Clinics Cologne, Women´s Clinic and Breast Center, Cologne, Germany

^9^West German Study Group, Moenchengladbach, Germany

^10^Breast Center, Department of Gynecology and Obstetrics and CCC Munich, LMU University Hospital, Munich, Germany

^11^Department of Gynecology, University Medical Center Hamburg, Hamburg, Germany

^12^Institute of Biostatistics and Registry Research, Brandenburg Medical School Theodor Fontane, Neuruppin, Germany

^13^Institute of Pathology, Medical School Hannover, Hannover, Germany

^14^German Center for Lung Research (DZL), BREATH, Hanover Germany

^¥^ share first authorship.

* Corresponding authors:

RWTH Aachen University Hospital, Pauwelsstraße 30, 52074 Aachen

Phone: +49-241-80-88431 (ED), +49-241-80-85248 (AS); Fax: +49-241-80-82439 (ED), +49-241-80-82008 (AS); Email: edahl@ukaaachen.de (ED), antonio.sechi@rwth-aachen.de (AS).

**Materials and Methods**

## TCGA analysis

Infinium HumanMethylation450 BeadChip data and Illumina HiSeq data from The Cancer Genome Atlas (TCGA) breast cancer cohort were analyzed for *SPAG6*. Promoter methylation of *SPAG6* was calculated using the mean value of the methylation of six CpGs (cg12610471, cg18247055, cg05099508, cg24031355, cg10648197, cg06908778) present in the *SPAG6* promoter (position -348 to +165)^1^.

## Patient population

Data from an additional and independent breast cancer cohort was provided by the West German Study Group (WSG). A tissue microarray (TMA) of their Phase III PlanB Trial that enrolled 3198 breast cancer patients between 2009 and 2011 addressing prognosis and prediction by the 21-gene Recurrence Score (RS) assay ^2^ was used. The PlanB trial was approved by German ethics boards and conducted following the Declaration of Helsinki.

## Production of lentivirus and cell infection

Stable overexpression was obtained by lentivirus-mediated gene delivery as previously described ^3, 4^. Infected cells were selected by fluorescence-activated cell sorting at the Flow Cytometry Facility, a core facility of the Interdisciplinary Centre for Clinical Research (IZKF) Aachen within the Faculty of Medicine at RWTH Aachen University.

## RNA isolation, cDNA synthesis and RT-PCR

Total RNA was isolated using the Nucleospin RNA plus kit (Macherey-Nagel, Düren, Germany), according to the manufacturer’s protocol. The RNA was eluted with 60 µl of nuclease-free water and measured spectrophotometrically with Nanodrop (Thermo Fisher Scientific). cDNA was synthesized using the reverse transcription system (Promega, Madison, USA) as described previously ^5^. cDNAs were amplified by semi-quantitative real-time PCR using the iTaq Universal SYBR green super mix (Bio-Rad Laboratories, Munich, Germany), performed in a CFX96 cycler (Bio-Rad Laboratories) according to standard procedures. All reactions were performed in triplicate. Primer sequences are listed in Table S11. All primers span an exon-intron boundary. Relative mRNA expression was calculated with the comparative CT (2^-ΔΔCq^) method and normalized to WT ^6^.

## Western blot

Cell lysates and western blotting were done as previously described^7^. Briefly, cultured cells were rinsed with ice-cold PBS solution and subsequently lysed in RIPA buffer that included proteinase and phosphatase inhibitors. The total protein concentration in each sample was assessed using the BCA assay, following the guidelines provided by the manufacturer. Equal quantities of total protein (30 μg) were subjected to standard SDS-PAGE electrophoresis, followed by transfer onto nitrocellulose membranes (0.2 µm, Schleicher & Schuell, Dassel, Germany). The primary antibodies used are detailed in Table S8-9. Visualisation of the primary antibodies was achieved through the application of horseradish-peroxidase (HRP)-conjugated secondary antibodies (Santa Cruz Biotechnology, Santa Cruz, CA, USA), employing the SuperSignal West Dura Extended Duration substrate (Perbio Science). The signal was detected by chemiluminescence (Thermo Fisher Scientific). β-actin antibody served as a loading control.

## Immunohistochemistry

Immunohistochemical analysis was performed according to the protocol described by Rose et al. ^7^ except that the EnVision FLEX Immunohisto Kit (Agilent, Santa Clara, CA, USA) was used. FFPE sections (3 µm) were incubated for 1 h at room temperature with SPAG6 antibody (HPA038440, Merck, Darmstadt, Germany) diluted at 1:200. Non-cancerous testicle tissue served as positive control and non-cancerous colon mucosa tissue served as negative control. ^8, 9^ SPAG6 protein staining was quantified by a certified pathologist using an adapted immunoreactive scoring system (IRS) according to Remmele and Stegner ^10^.

## Immunolabeling

Immunolabelling was done as previously described with minor changes ^3, 4^. After culturing T-47D and MCF-7 cells on μ-Dishes (Ibidi, Martinsried, Germany) for 24 hours, cells were fixed using different fixation protocols depending on the antibodies or antibody combinations (Tables S7, S8 and S9). Cell nuclei were stained with DAPI. Images were collected by confocal microscopy using an LSM 700 confocal system (Zeiss) equipped with a 63x/1.3 NA objective and 350, 488, 555 and 635 nm laser lines.

## Wound-healing assay

Wound healing assays were done as already described ^3^. Briefly, 1 x 10^5^ cells were plated in silicon inserts (Ibidi, Martinsried, Germany) mounted on μ-Dishes (Ibidi) and incubated overnight at 37°C, 5% CO_2_. Cell proliferation was inhibited by treating the cells with 10 µg/ml mitomycin C for 30 minutes before starting the assay. Cell migration was recorded for 24 hours using an Axio observer Z1 inverted microscope equipped with a heating stage, CO_2_ controller and an Evolve EM-CCD camera driven by ZEN software (Zeiss, Jena, Germany). Average cellular speed was calculated by measuring the distance (at equally spaced locations along the longest dimension of the wound) travelled by the wound edge before closing the wound ^3^.

**Table S1. Clinicopathological parameters of the TCGA cohort.**

|  | N (%) |
| --- | --- |
| Age at diagnosis |  |
| median age | 58 |
| ≤ median | 607 (53) |
| > median | 549 (47) |
| Unknown | 0 (0) |
| Histological type |  |
| IDC | 842 (73) |
| ILC | 195 (17) |
| Other | 117 (10) |
| Unknown | 2 (1) |
| Tumor size |  |
| pT1 | 291 (25) |
| pT2 | 677 (59) |
| pT3 | 141 (12) |
| pT4 | 40 (3) |
| Unknown | 7 (1) |
| Lymph node status |  |
| pN0 | 535 (46) |
| pN1 | 392 (34) |
| pN2 | 127 (11) |
| pN3 | 76 (6) |
| Unknown | 26 (2) |
| ER |  |
| Positive | 560 (48) |
| Negative | 172 (15) |
| Unknown | 424 (37) |
| PR |  |
| Positive | 487 (42) |
| Negative | 242 (21) |
| Unknown | 427 (37) |
| HER2 |  |
| Positive | 103 (9) |
| Negative | 619 (54) |
| Unknown | 434 (38) |

Percentages may not sum up to 100% due to rounding.

**Table S2. Clinicopathological parameters of the West German Study Group Phase III PlanB Trial cohort from all patients with available SPAG6 measurements (N = 2241).**

|  | N (%) |
| --- | --- |
| Histological type |  |
| IDC | 1903 (85) |
| ILC | 242 (11) |
| Other | 88 (4) |
| Unknown | 1903 (85) |
| Luminal A |  |
| No | 1270 (60) |
| Yes | 851 (40) |
| Missing | 120 (5) |
| Luminal B |  |
| No | 1234 (58) |
| Yes | 881 (42) |
| Missing | 126 (6) |
| TNBC |  |
| No | 1807 (83) |
| Yes | 379 (17) |
| Missing | 55 (2) |
| Age / years |  |
| Mean (SD) | 55.39 (10.03) |
| ER / % |  |
| Mean (SD) | 74.88 (39.43) |
| Missing | 58 (3) |
| PR / % |  |
| Mean (SD) | 51.10 (44.48) |
| Missing | 70 (3) |
| HER2 / % |  |
| Mean (SD) | 8.92 (0.80) |
| Missing | 413 (18) |
| Ki67 |  |
| Mean (SD) | 20.45 (16.53) |
| Missing | 118 (5) |

Percentages for categories other than missing are obtained among non-missing observations and they sum up to 100%.

**Table S3. Multivariable Cox models for OS.** The following variables were selected for multivariable models based on univariable models: composite nodal status, treatment group, luminal A, TN, ER, PR, HR, age, histological grade, composite tumor stage, and Ki67. Moreover, TN, ER, HR, histological grade, and Ki67 were confounders. However, since luminal A, TN, and HR were associated with ER and PR, only the last two were considered for multivariable models.

| **Characteristic** | **Label** | **N patients** | **N events** | **HR [95%CI]** | **p-value** |
| --- | --- | --- | --- | --- | --- |
| SPAG6 | Negative | 1450 | 72 | 1 |  |
|  | Positive | 508 | 22 | 0.92 [0.57, 1.49] | 0.727 |
| Composite nodal status | 0 | 1178 | 43 | 1 |  |
|  | 1 | 659 | 36 | 2.43 [1.53, 3.86] | <0.001 |
|  | 2-3 | 121 | 15 | 4.80 [2.61, 8.85] | <0.001 |
| PR status | Negative | 648 | 58 | 1 |  |
|  | Positive | 1310 | 36 | 0.50 [0.31, 0.82] | 0.006 |
| Age | <=50 | 681 | 23 |  |  |
|  | >50 | 1277 | 71 | 1.85 [1.15, 3.00] | 0.012 |
| Histological grade | 0-2 | 1094 | 23 | 1 |  |
|  | 3 | 864 | 71 | 2.33 [1.36, 4.01] | 0.002 |
| Composite tumor stage | 1 | 1066 | 35 | 1 |  |
|  | 2-4 | 892 | 59 | 1.62 [1.06, 2.48] | 0.027 |
| Ki67 (per 10%) |  | 1958 | 94 | 1.27 [1.15, 1.42] | <0.001 |

**Table S4. Multivariable Cox models for DFS.** Variables selected for multivariable models based on univariable models were composite nodal status, treatment group, luminal A, TN, ER, PR, HR, histological grade, composite tumor stage, and Ki67. Moreover, luminal A, TN, ER, PR, HR, histological grade, and Ki67 were confounders. However, since luminal A, TN, and HR were associated with ER and PR, only the last two were considered for multivariable models. Final multivariable models contained dichotomized SPAG6 expressions (negative, positive) and all significant prognostic factors and confounders.

| **Characteristic** | **Label** | **N patients** | **N events** | **HR [95%CI]** | **p-value** |
| --- | --- | --- | --- | --- | --- |
| SPAG6 | Negative | 1450 | 146 | 1.00 |  |
|  | Positive | 508 | 47 | 1.02 [0.73, 1.42] | 0.927 |
| Composite nodal status | 0 | 1178 | 101 | 1.00 |  |
|  | 1 | 659 | 63 | 1.59 [1.15, 2.21] | 0.005 |
|  | 2-3 | 121 | 29 | 3.91 [2.55, 5.99] | <0.001 |
| PR status | Negative | 648 | 104 | 1.00 |  |
|  | Positive | 1310 | 89 | 0.58 [0.42, 0.80] | 0.001 |
| Histological grade | 0-2 | 1094 | 63 | 1.00 |  |
|  | 3 | 864 | 130 | 1.80 [1.26, 2.56] | 0.001 |
| Composite tumor stage | 1 | 1066 | 77 | 1.00 |  |
|  | 2-4 | 892 | 116 | 1.58 [1.18, 2.12] | 0.002 |
| Ki67 (per 10%) |  | 1958 | 193 | 1.20 [1.11, 1.30] | <0.001 |


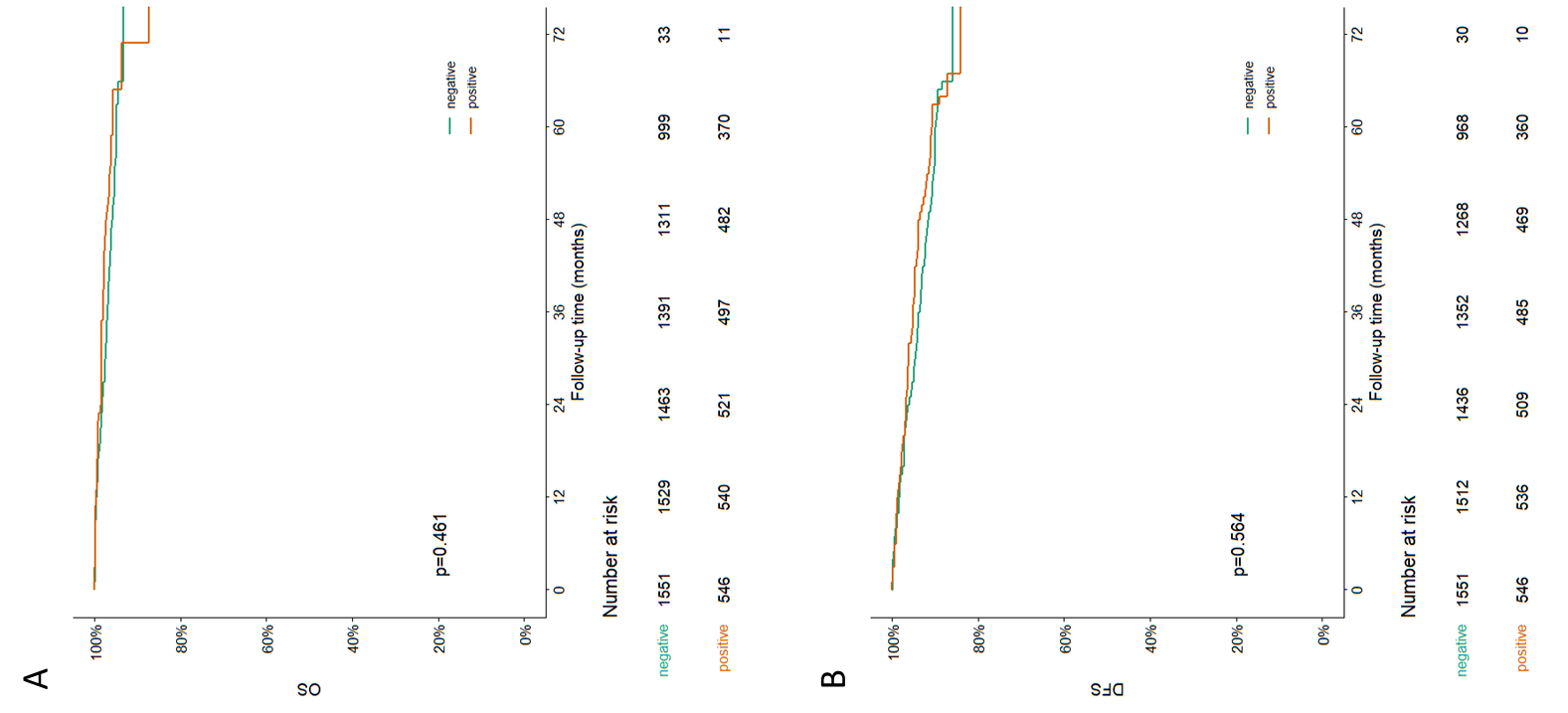


**Figure S1. Kaplan-Meier curves and p-values of log-rank test between negative and positive SPAG6 expressing breast cancer patients of the WSG Phase III PlanB Trial cohort.** Overall survival (A), disease-free survival (B).

**Table S5. Adjusted interaction model of SPAG6 effects in luminal A for DFS.** Cox model adjusted for composite nodal status, histological grade, composite tumor stage, Ki67 with p-value of HR and p-interaction between HR of Luminal A negative and positive.

| **Variable** | | **HR [95%CI]** | **p-value** | **p-interaction** |
| --- | --- | --- | --- | --- |
| Luminal A negative | SPAG6 negative | 1.00 |  | 0.085 |
|  | SPAG6 positive | 0.86 [0.58, 1.28] | 0.463 |  |
| Luminal A positive | SPAG6 negative | 1.00 |  |  |
|  | SPAG6 positive | 1.64 [0.88, 3.06] | 0.116 |  |

**Table S6. Adjusted interaction model of SPAG6 effects in luminal A for OS.** Cox model adjusted for composite nodal status, age, histological grade, composite tumor stage, Ki67 with p-value of HR and p-interaction between HR of Luminal A negative and positive.

| **Variable** | | **HR [95%CI]** | **p-value** | **p-interaction** |
| --- | --- | --- | --- | --- |
| Luminal A negative | SPAG6 negative | 1.00 |  | 0.819 |
|  | SPAG6 positive | 0.88 [0.51, 1.49] | 0.627 |  |
| Luminal A positive | SPAG6 negative | 1.00 |  |  |
|  | SPAG6 positive | 1.02 [0.32, 3.20] | 0.978 |  |


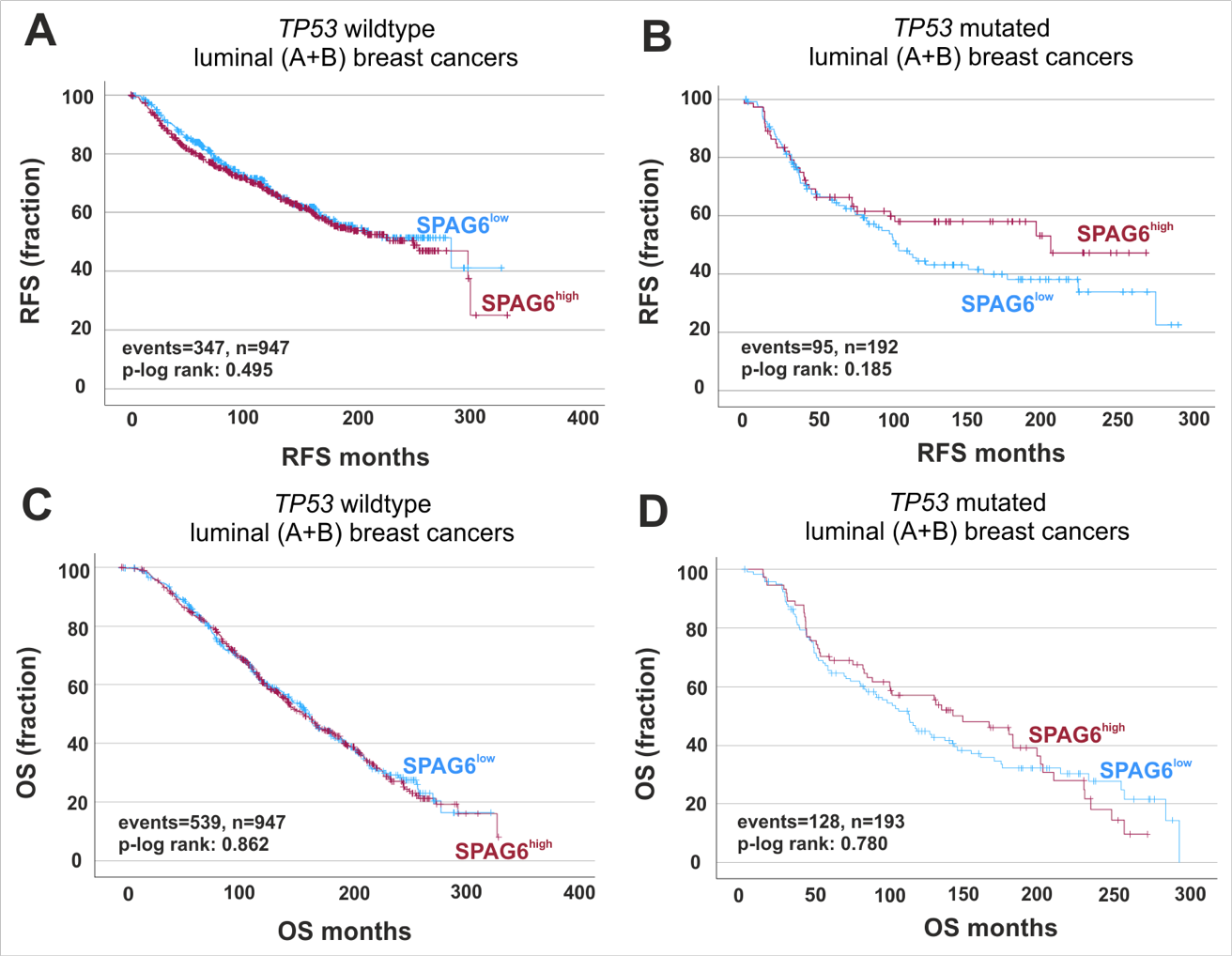


**Figure S2. Kaplan-Meier curves and p-values showing the relationship between SPA6 expression and TP53 mutation status on patient prognosis.** Recurrence-free survival (RFS) (A, B) and overall survival (OS) (C, D) parameters for luminal A and luminal B breast cancers expressing high (red curves) or low (blue curves) level of SPAG6, categorised by the expression of either wild-type TP53 (A, C) or mutated TP53 (B, D).


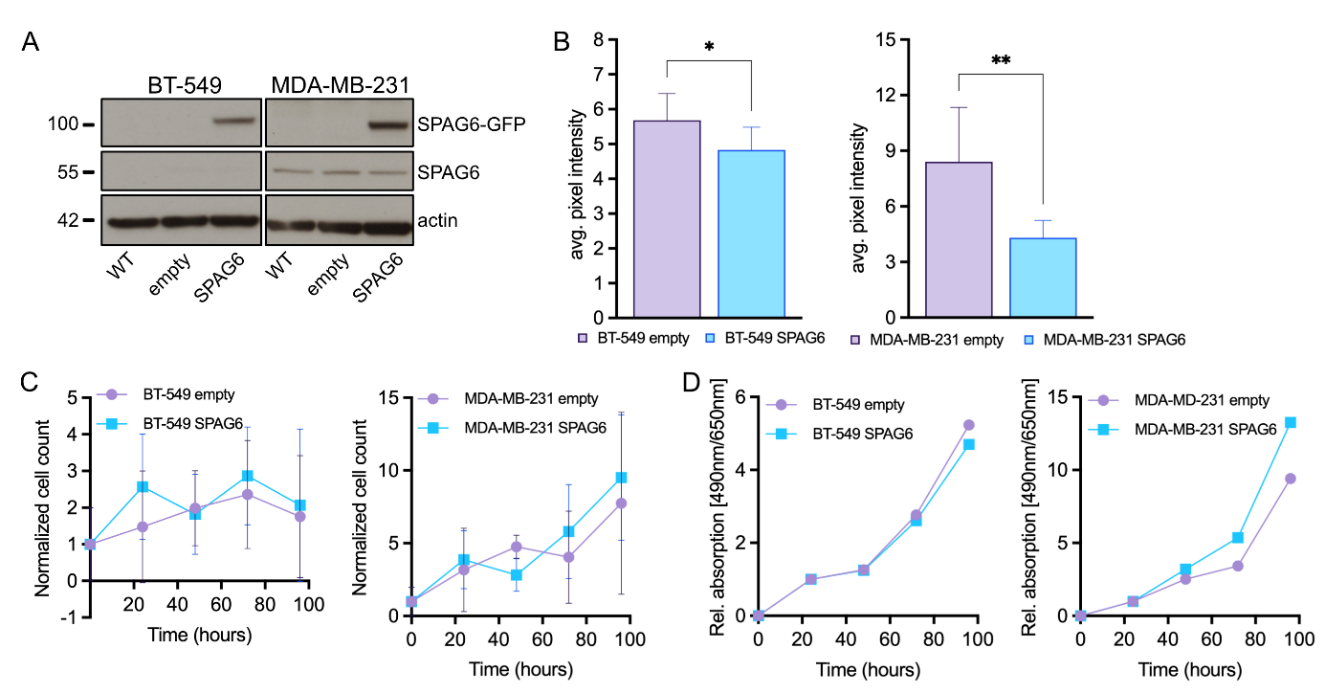


**Figure S3. Preliminary analysis of basal-type breast cancer cell lines overexpressing SPAG6.** (A) Expression of endogenous and GFP-tagged SPAG6, (B) Data summary from colony formation assay, (C), Cell counting assay and (D) XTT proliferation assay.

**Table S7. Fixation and staining protocols.**

|  | Fixation | | Staining | |
| --- | --- | --- | --- | --- |
|  | **1. step** | **2. step** | **Primary antibody*** | **Secondary antibody^#^** |
| β-actin | 1% PFA%/0,5% Tx-100  (15 min at RT) | 4% PFA  (10 min at RT) | - | Phallodin-594 |
|  |  |  |  | DAPI (1:1000) |
| β-catenin | 1% PFA%/0,5% Tx-100  (15 min at RT) | 4% PFA  (10 min at RT) | anti-β-catenin (1:200) | Anti-mouse IgG-Alexa Fluor 594 (1:500) |
|  |  |  |  | DAPI (1:1000) |
| E-cadherin | 1% PFA%/0,5% Tx-100  (15 min at RT) | 4% PFA  (10 min at RT) | anti-E-cadherin (1:200) | Anti-rabbit IgG-Alexa Fluor 647 (1:500) |
|  |  |  |  | DAPI (1:1000) |
| Tubulin | 4% PFA  (20 min at RT) | 0,1% TX-100 in PBS (1 min at RT) | anti-YL1/2 Tubulin | Anti-rat IgG-Alexa Fluor 647 (1:500) |
|  |  |  |  | DAPI (1:1000) |
| Vimentin | Pre-chilled methanol  (-20°C) and cells on ice | - | anti-vimentin (1:100) | Anti-rabbit IgG-Alexa Fluor 647 (1:500) |
|  |  |  |  | DAPI (1:1000) |
| Vinculin | 1% PFA%/0,5% Tx-100  (15 min at RT) | 4% PFA  (10 min at RT) | anti-vinculin (1:200) | Anti-mouse IgG-Alexa Fluor 594 (1:500) |
|  |  |  |  | DAPI (1:1000) |

* Diluted in 1% BSA in TBS, 30-minutes incubation at RT; ^#^ Diluted in 1% BSA in TBS, 30-minutes incubation at RT in the dark; RT: room temperature

**Table S8.** **List of primary antibodies.**

| Antibody | Clonality | Host | Manufacturer | Dilution |
| --- | --- | --- | --- | --- |
| anti-ß-actin | monoclonal | mouse | Sigma-Aldrich, Missouri, USA | 1:2000 |
| anti-ß-catenin | monoclonal | mouse | Proteintech, Rosemont, USA | 1:200 |
| anti-E-cadherin | polyclonal | rabbit | Proteintech, Rosemont, USA | 1:200 |
| anti-tubulin | monoclonal | rat | Own production by hybridoma | undiluted |
| anti-SPAG6 | polyclonal | rabbit | Sigma-Aldrich, Missouri, USA | 1:250 |
| anti-vimentin | polyclonal | rabbit | Proteintech, Rosemont, USA | 1:100 |
| anti-vinculin | monoclonal | mouse | Sigma-Aldrich, Missouri, USA | 1:200 |

**Table S9.** **List of secondary antibodies.**

| Antibody | Clonality | Host | Manufacturer | Dilution |
| --- | --- | --- | --- | --- |
| anti-mouse IgG | polyclonal | goat | Dako, Glostrup, Denmark | 1:8000 |
| anti-rabbit IgG | polyclonal | goat | Dako, Glostrup, Denmark | 1:10.000 |
| anti-mouse IgG Alexa Fluor 594 | polyclonal | goat | Life technologies, Carlsbad, USA | 1:500 |
| anti-rabbit IgG Alexa Fluor 647 | polyclonal | goat | Life technologies, Carlsbad, USA | 1:500 |
| anti-rat IgG Alexa Fluor 647 | polyclonal | goat | Life technologies, Carlsbad, USA | 1:500 |

**Table S10.** **Primers used for SPAG6 cloning.**

| Primer name | Primer sequence (5' 🡪 3') |
| --- | --- |
| SPAG6_BamHI_For | TCAGGGATCCATGAGTCAGAGGCAG |
| SPAG6_MluI_Rev | CTGAACGCGTTTGTTATTAAGTGGTTGATA |

**Table S11.** **Primers used for real-time PCR and their annealing temperature (TA).**

| Primer name | | Primer sequence (5' 🡪 3') | Cycles | TA |
| --- | --- | --- | --- | --- |
| *ß-actin* | Forward | TGACGTGGACATCCGCAAAG | 40 | 60˚C |
|  | Reverse | CTGGAAGGTGGACAGCGAGG |  |  |
| *SPAG6* | Forward | AGGGTGTACCCCAGTTGTCA | 40 | 60˚C |
|  | Reverse | TTTTTACTTTTTACTTGGAGATCCTCAGAA |  |  |
| *E-cadherin* | Forward | TGCCCAGAAAATGAAAAAGG | 40 | 60˚C |
|  | Reverse | GTGTATGTGGCAATGCGTTC |  |  |
| *Vimentin* | Forward | TCCACGAAGAGGAAATCCAG | 40 | 60˚C |
|  | Reverse | TTCCAGGGACTCATTGGTTC |  |  |

**Legend for supplementary videos**

**Video S1.** Wound healing assay. Control MCF7 cells (left side) and MCF7 cells stably expressing SPAG6 (right side) were filmed for 24 hours at 37°C, 5% CO_2_. Images were acquired every 5 minutes. The playback speed is approximately 6.000 times the original speed.

**Video S2.** Wound healing assay. Control T47D cells (left side) and T47D cells stably expressing SPAG6 (right side) were filmed for 24 hours at 37°C, 5% CO_2_. Images were acquired every 5 minutes. Note the significantly faster motility of cells expressing SPAG6. The playback speed is approximately 6.000 times the original speed.

**References**

1 Shen Y, Chow J, Wang Z, Fan G. Abnormal CpG island methylation occurs during in vitro differentiation of human embryonic stem cells. *Hum Mol Genet*. 2006; 15: 2623-2635.

2 Gluz O, Nitz UA, Christgen M, et al. West German Study Group Phase III PlanB Trial: First Prospective Outcome Data for the 21-Gene Recurrence Score Assay and Concordance of Prognostic Markers by Central and Local Pathology Assessment. *J Clin Oncol*. 2016; 34: 2341-2349.

3 Gamper I, Fleck D, Barlin M, et al. GAR22beta regulates cell migration, sperm motility, and axoneme structure. *Mol Biol Cell*. 2016; 27: 277-294.

4 Maxeiner S, Shi N, Schalla C, et al. Crucial role for the LSP1-myosin1e bimolecular complex in the regulation of Fcgamma receptor-driven phagocytosis. *Mol Biol Cell*. 2015; 26: 1652-1664.

5 Veeck J, Chorovicer M, Naami A, et al. The extracellular matrix protein ITIH5 is a novel prognostic marker in invasive node-negative breast cancer and its aberrant expression is caused by promoter hypermethylation. *Oncogene*. 2008; 27: 865-876.

6 Livak KJ, Schmittgen TD. Analysis of relative gene expression data using real-time quantitative PCR and the 2(-Delta Delta C(T)) Method. *Methods*. 2001; 25: 402-408.

7 Rose M, Kloten V, Noetzel E, et al. ITIH5 mediates epigenetic reprogramming of breast cancer cells. *Mol Cancer*. 2017; 16: 44.

8 Neilson LI, Schneider PA, Deerlin PGV, et al. cDNA Cloning and Characterization of a Human Sperm Antigen (SPAG6) with Homology to the Product of the Chlamydomonas PF16 Locus. *Genomics*. 1999; 60: 272-280.

9 Zhang Z, Sapiro R, Kapfhamer D, et al. A sperm-associated WD repeat protein orthologous to Chlamydomonas PF20 associates with Spag6, the mammalian orthologue of Chlamydomonas PF16. *Mol Cell Biol*. 2002; 22: 7993-8004.

10 Remmele W, Stegner HE. [Recommendation for uniform definition of an immunoreactive score (IRS) for immunohistochemical estrogen receptor detection (ER-ICA) in breast cancer tissue]. *Pathologe*. 1987; 8: 138-140.
